# Study on Preparation Process of Anticoagulant BAY2433334

**DOI:** 10.3390/molecules29246039

**Published:** 2024-12-21

**Authors:** Yanqun Zeng, Guodong Cen, Guanglin Zhou, Xucheng Zhu, Long Huang, Xiaoyu Wang

**Affiliations:** Chengdu Shibeikang Biomedical Technlogy Co., Ltd., 26-1-2, No.2 Tianyu Road, Chendu Gaoxin West District, Chengdu 611700, China

**Keywords:** BAY2433334, (2*R*)-2-aminobutyric acid, *p*-toluenesulfonyl chloride, N-alkylation/O-alkylation ratio, ee value

## Abstract

A new process route suitable for the industrial production of BAY2433334 has been developed in this paper, which avoids the patent limitations of the originator company of BAY2433334 to the preparation of BAY2433334. BAY2433334 is obtained from (2*R*)-2-aminobutyric acid by esterification, diazotization, condensation reactions, deacetyl deprotection, activation reactions, and Mitsunobu reactions. This method is simple to operate, and the raw materials are inexpensive and readily available. Simultaneously, the product quality is very high; few O-alkylated impurities are generated during the reaction, with a high N-alkylated product/O-alkylated product ratio (above 35–45:1). As a result, the ee value is greater than 99%, which means that there are very few isomers present such that no chiral resolution is required, which greatly reduces the cost.

## 1. Introduction

With population aging and changes in people’s lifestyles and habits, thromboembolic diseases are increasingly becoming a major global health problem and the leading cause of death worldwide [1]. Thromboembolic disease is a condition in alive humans and animals caused by abnormal blood clots formed within blood vessels. Atrial fibrillation can lead to thromboembolic events, which carry a high risk of permanent disability and death [2,3]. Treatment guidelines [4,5] recommend the use of oral anticoagulation in patients with atrial fibrillation, preferably with direct-acting oral anticoagulants (DOACs) owing to their greater safety and efficacy as compared with those of vitamin K antagonists. But many patients do not receive anticoagulants, owing to their anticipated risk of bleeding or the occurrence of actual bleeding events [6,7].

FXI (coagulation factor XI) is part of the intrinsic pathway of the coagulation cascade activated by FXII (coagulation factor XII). FXIa (coagulation factor XIa) is a plasma serine protease, primarily synthesized by hepatocytes and circulates in the zymogen form (FXI) [8]. Unlike the final common pathway involving coagulation factors like FX that lead to the activation of thrombin, FXIa plays a minor role in the physiological hemostatic mechanisms activated after local vascular injury [9]. The activation of the endogenous pathway must meet specific physiological conditions, including in vitro contact activation with negatively charged molecules such as dextran sulfate and silica and the in vivo release of negatively charged genomic substances in the form of NETs (neutrophil extracellular traps). The main positive feedback mechanism for the sustained activation of FXIa is the interaction with circulating thrombin during the amplification phase. Most persons with factor XI deficiency do not have spontaneous bleeding, hemarthroses, or hematomas and have a lower incidence of cardiovascular events, especially cardioembolic stroke, than persons without FXI deficiency [10,11]. Severe FXI deficiency (10–20% of normal levels) can prevent venous thrombosis and reduce the incidence of thrombosis [10]. Based on these physiological coagulation mechanisms, directly inhibiting FXIa or inhibiting the production of FXIa or dysfunction, theoretically, has great potential to produce effective antithrombotic effects without sacrificing its protective role in hemostasis [12]. Therefore, drugs targeting FXIa can block the endogenous pathway and inhibit the amplification of the coagulation cascade, thereby exerting antithrombotic effects.

In recent years, studies have shown that the inhibition of FXIa may result in a lower bleeding risk compared to direct FXa inhibitors. Thus, FXIa is a new target for antithrombotic prevention and treatment. Mechanisms to induce functional FXIa deficiency include inhibiting the biosynthesis of FXIa using antisense oligonucleotides (ASOs) or directly inhibiting FXIa signaling pathways with small peptide mimetic molecules, monoclonal antibodies, aptamers, or natural inhibitors. Although drugs like IONIS FXI-LRx (ISIS 416858) ASO [13] improve anticoagulant effects over a long time by inhibiting the biosynthesis of FXIa, small-molecule FXIa inhibitors such as BAY2433334 [14] are preferred clinically because these inhibitors have characteristics of “rapid onset and rapid elimination” and, theoretically, are suitable for patients who need to temporarily interrupt chronic anticoagulation therapy due to bleeding, critical care, or perioperative conditions. There is an unmet need for effective anticoagulation that prevents stroke but with a lower risk of bleeding among patients. Compared to direct oral anticoagulants (DOACs), such as direct thrombin inhibitors (dabigatran) and factor Xa inhibitors (apixaban, rivaroxaban, and edoxaban), FXIa inhibitors may be more suitable for the prevention of arterial or venous thrombosis indications.

BAY2433334 is a novel oral small-molecule FXIa inhibitor developed by Bayer, Germany [14], that is administered orally once daily and has a mean terminal half-life of 16 to 18 h with less than 15% renal elimination [15,16]. The results of the Phase II clinical trial PACIFIC-AF released in 2022 showed that BAY2433334 50 mg qd could inhibit over 90% of XIa activity, and compared to apixaban, the bleeding risk was significantly reduced [17]. In 2022, BAY2433334 was qualified for fast channel by the FDA for secondary prevention in patients with non-cardioembolic ischemic stroke. In May 2023, BAY2433334 was qualified for fast channel again by the FDA as a potential therapy for the prevention of stroke and systemic embolism in patients with atrial fibrillation. Currently, multiple Phase III clinical trials are being conducted globally, although the Phase III clinical trial OCEANIC-AF, aimed at preventing stroke or systemic embolism in patients with atrial fibrillation, was terminated early due to insufficient efficacy compared to Eliquis [18]. However, another clinical trial, OCEANIC-STROKE, is ongoing. This trial aims to evaluate the performance of BAY2433334 in combination with standard antiplatelet therapy in the prevention of ischemic stroke. It is planned to enroll 12,300 patients and be completed in October 2025. Therefore, BAY2433334 is still expected to become a new-generation anticoagulant with low bleeding risk. BAY2433334 has a relatively complex structure, making synthesis challenging. Its isomers are difficult to separate, scale-up production is extremely challenging, and it is limited by patent protection. Therefore, the synthesis process study is of great significance.

## 2. Analysis and Design of Synthetic Route

Patents WO2014154794 [19] and WO2017005725 [20] disclose the preparation methods for a series of compounds such as BAY2433334. Using 2,5-dimethoxypyridine as the starting raw material, the target compounds are synthesized in nine steps employing a linear synthesis strategy, which causes a lengthy route and high probability of significant racemization, resulting in low overall yield. In addition, cumbersome post-treatment and purification procedures, such as the separation of isomers by HPLC or chiral supercritical fluid chromatography (SFC), are required, which is time-consuming and expensive, unsuitable for industrial scale-up production.

**Figure 1:**
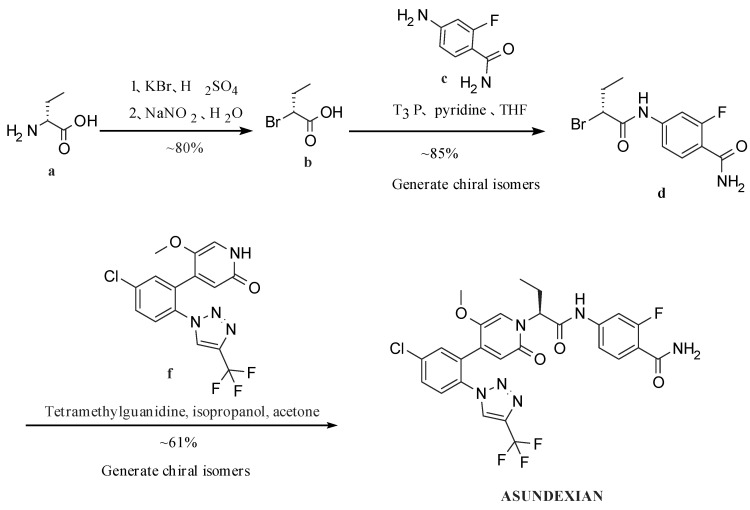
Synthetic route reported in CN111770917A.

**Figure 2:**
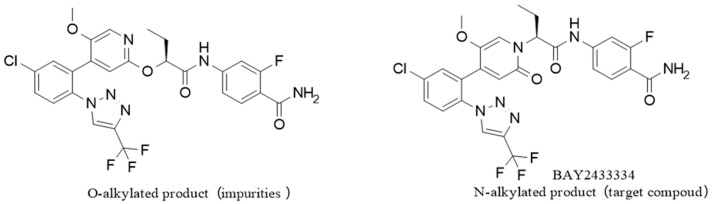
Structure of O-alkylation product and N-alkylation product.

Patent CN111770917A [21] discloses a convergent synthesis strategy. The steps for synthesizing the crude product are shown in Figure 1 below. After critical intermediates formula f compound and formula d compound are synthesized, respectively, the crude product of BAY2433334 is generated by a condensation reaction. Six steps are performed for the total reaction, which shortens the reaction period and focuses on optimizing the enantioselectivity and N/O-alkylation selectivity (Figure 2) of the crude synthesis step. The condensate is filtered and evaporated to obtain the amorphous form of crude BAY2433334, with an enantiomeric excess value (abbreviated as the ee value) reaching 85% to 93% and the ratio of the N-alkylation product to O-alkylation product reaching 9:1 to 10:1.

Although the convergent synthesis route of this patent is overall superior to the linear synthesis strategy, its condensation reaction in crude product synthesis still has significant limitations, such as the following:

(1) Low conversion: Yields of the substitution reaction in the crude synthesis of BAY2433334 were 70% and 75% only. The alternative method had a yield as low as 61% (paragraphs 0095-0097 in the labeling), and the total yield over six steps was only 20–25% (paragraph 0054 in the labeling) [21].

(2) High proportion of isomers: Although the ee value of the crude product has been optimized to 85–93%, 7–15% of isomer impurities were present. Moreover, further purification with organic solvents was required to obtain a purified crude product with a >99% ee value, and then the target crystal was obtained through a crystallization process.

(3) Poor positional selectivity of N/O-alkylation: Although the ratio of crude N-alkylated product to O-alkylated product reached (9–10):1, the O/N conversion (about 10%) was undesirable, which not only led to low conversion and increased costs but also produced a large quantity of O-alkylated product, as shown in Figure 2. These were difficult to purify and remove during post-treatment and would reduce product quality in that case.

Considering the above factors, the subsequent purification difficulty and product quality control risk of BAY2433334 remain urgent issues to be resolved. Therefore, how to improve the product quality of the target compound, reduce impurity control risk, increase product conversion and purity, shorten the production cycle, reduce costs, and make it more suitable for industrial scale-up production is a technical problem that urgently needs to be solved in this field.

**Figure 3:**
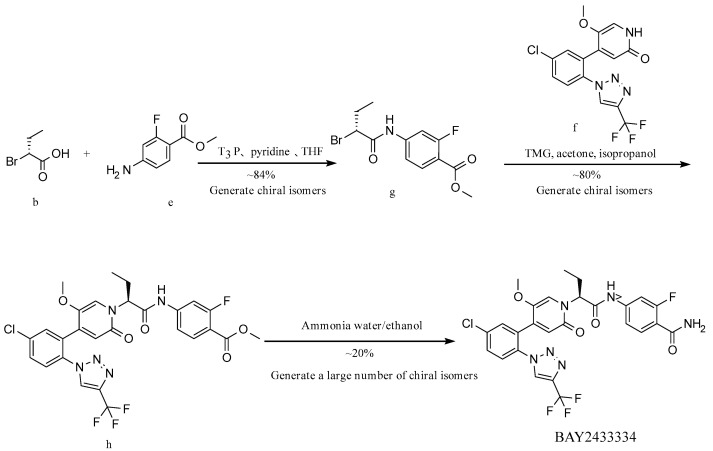
Synthesis Route 1.

Our synthetic laboratory designed four different synthetic routes and conducted studies accordingly. The results are as follows:

**Route 1 (Figure 3):**

In this route (Figure 3), at first, intermediate g is generated from intermediate b and material e by a condensation reaction. Then, intermediate g reacts with raw material f to obtain intermediate h. Finally, intermediate h and ammonia are transesterified under alkaline conditions to obtain the target BAY2433334. This route is similar to the reaction route disclosed in patent CN 111770917 A and has the same issues, in which a large proportion of the resulting product racemizes, since the transesterification occurs under basic conditions, leading to low yield and a high proportion of isomers.

**Figure 4:**
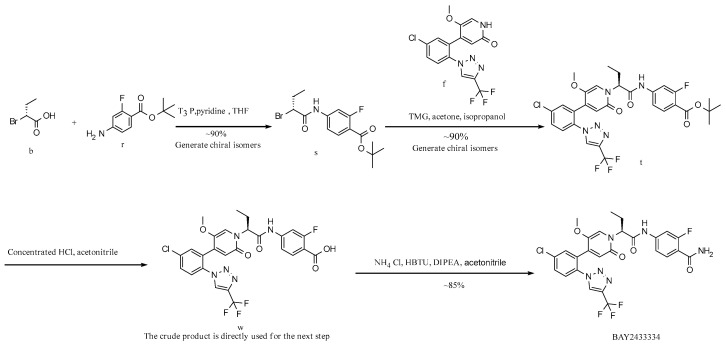
Synthesis Route 2.

**Route 2 (Figure 4):**

In this route (Figure 4), at first, intermediate s is generated from intermediate b and material r by a condensation reaction, with a yield of approximately 90%; intermediate s reacts with raw material f to obtain intermediate t, with a yield of about 90%; intermediate w is generated from intermediate t by de-tert-butylation, and the crude product is directly used in the next reaction; the target BAY2433334 is synthesized from intermediate w and ammonium chloride by a condensation reaction, with a column yield of about 85%. Similarly to Route 1, although the overall yield of this route is relatively high, there is a possibility of racemization at the carbonyl α position of the amide bond in the first and second steps of the reaction. In addition, the raw material f is expensive, which results in higher synthesis costs when it is used in the second step of this route.

**Figure 5:**
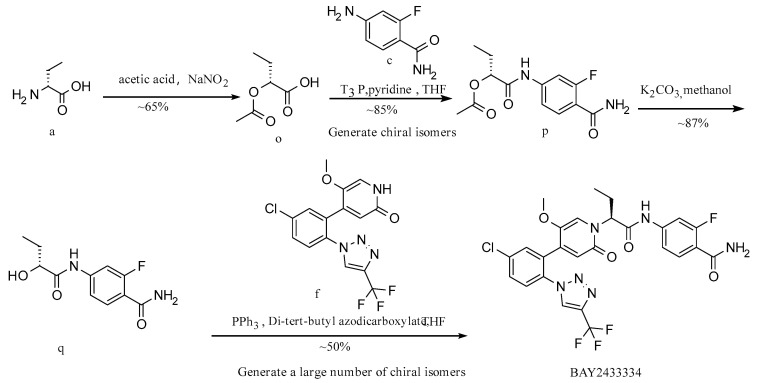
Synthesis Route 3.

**Route 3 (Figure 5):**

In this route (Figure 5), at first, intermediate o is generated from raw material a by a diazotization reaction. Intermediate p is generated from intermediate o and raw material c by a condensation reaction; intermediate q is generated from intermediate p by deacetylation. Eventually, the product BAY2433334 is synthesized from intermediate q and raw material f by a Mitsunobu reaction. The reaction selectivity of the last step is poor, there are many impurities, and the yield is low.

**Figure 6:**
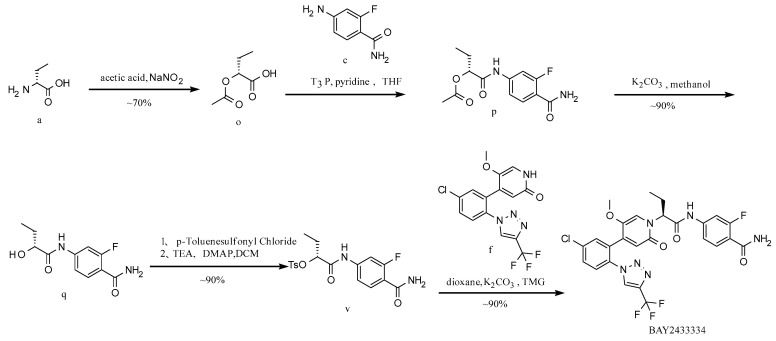
Synthesis Route 4.

**Route 4 (Figure 6):**

In this route (Figure 6), at first, intermediate o is generated from raw material a by a diazotization reaction; intermediate p is generated from intermediate o and raw material c by a condensation reaction; intermediate q is generated from intermediate p by deacetylation; intermediate q reacts with *p*-toluenesulfonyl chloride to obtain intermediate v [22]; intermediate v reacts with raw material f to obtain the target BAY2433334. In this route, the expensive raw material f participates in the reaction in the last step, resulting in high yield, low cost, and a stable chiral configuration. Other raw materials are readily available on the market, and the synthetic route is relatively simple and suitable for subsequent industrial scale-up production.

## 3. Results and Discussion

In the preparation of intermediate o, different reaction conditions were attempted. Studies were conducted on conditions such as the feed ratio in the reaction, added with or without H_2_O, and post-treatment methods. The results are shown in Table 1. The yield after purity correction of the sodium nitrite/H_2_O/acetic acid reaction system was not high. Since the system contained H_2_O, it was speculated that hydroxyl by-products were generated. By adopting a H_2_O-free process for the reaction system, the yield increased upon scale-up production. Because the product is freely soluble in H_2_O and acetic acid, it was necessary to thoroughly remove acetic acid during post-treatment, and then the acidity was adjusted for extraction. The operation was complex, and the yield decreased after post-treatment. By further optimizing the post-treatment process, removing the step of water washing, directly concentrating, and adding dichloromethane for stirring, followed by filtration and concentration, the yield was significantly improved.

In the preparation of intermediate p, the effects of different condensing agents, solvents, catalysts, and bases on the reaction were investigated, as shown in Table 2. When CDI and EDCI were used as condensing agents, the purity of the reaction system was poor. When an acyl chlorination reaction was used, the effect of the feed ratio was investigated. It was found that when the molar ratio of raw material c to intermediate q was 1:1.7, the yield of crude product after purity correction was relatively high. However, upon considering the post-treatment methods, it was found that purification was not possible, with the purity only around 90%. When Propyl phosphoric anhydride (T3P) was used as the catalyst and pyridine was used as the base, as well as recrystallization in mixed solvents being employed for purification, a qualified white product with a high ee value and high purity was obtained. Moreover, by using intermediate p with an ee value of more than 99% to synthesize the target compound BAY2433334, the ee value of BAY2433334 was also more than 99%, which could greatly reduce the formation of isomers and increase the yield.

In the preparation of intermediate q, the effect of different bases on the reaction was investigated. The bases were studied to determine the most suitable base, and the results are shown in Table 3. Using potassium carbonate and cesium carbonate as bases, their strong basicity resulted in more impurities in the reaction. Using sodium carbonate as a base, fewer impurities were observed in the reaction. Therefore, sodium carbonate was selected as the base.

The preparation of intermediate v involved the investigation of the reaction temperature, as shown in Table 4. Lower temperatures were unfavorable for the reaction to proceed, while a reaction temperature of 30 ± 5 °C was more suitable, at which the reaction of raw material q was complete. Further increasing the temperature would increase the risk of impurities and energy consumption. Taking these factors into account, a reaction temperature of 30 ± 5 °C was selected.

Since the final prepared target compound BAY2433334 had a relatively high content, the initial drying temperature had a significant impact on the quality of the prepared target compound. Therefore, an investigation of the drying temperature was conducted. As shown in Table 5, higher initial drying temperatures (greater than 50 °C) caused the wet product to melt into lumps, and thus a qualified solid form could not be obtained. When drying at an initial temperature of 30–40 °C, the sample did not melt and the drying effect was good. Therefore, 30–40 °C was adopted as the initial drying temperature.

In addition, Route 4, adopted in this paper, has absolute advantages in improving total conversion (Table 6), enantioselectivity, and N/O-alkylation selectivity, especially in the selection of milder bases and more readily available solvents. It can greatly enhance positional isomer selectivity, increase the N-alkylation/O-alkylation ratio, and achieve an N/O ratio of over 35–45:1, which is beneficial for subsequent crystallization yield and improving the stability of crystallization purification process control.

Therefore, this route can prepare the target compound BAY2433334 with a high ee value by chemical methods alone, which solves the problem that the target compound BAY2433334 with a high ee value cannot be synthesized by the existing techniques, and it makes the chemical resolution method of BAY2433334 applicable in process scale-up without the need for expensive chiral supercritical fluid chromatography (SFC) resolution. Moreover, due to the reduction in isomers, the use of intermediate f is decreased, which is helpful to improve the quality of products at a lower cost and to win in the market. In summary, this is beneficial for the laboratory synthesis and industrial production of this new type of FXIa inhibitor molecule.

## 4. Materials and Methods

The liquid chromatography–mass spectrometry (LC-MS) instrument used is Agilent G6120B (Brand: Agilent, Origin, Germany) (paired with liquid chromatography Agilent 1260); the nuclear magnetic resonance spectrometer (^1^H NMR) is Bruker AVANCE-400 (Brand: Bruker, Origin, Germany). The ^1^H NMR chemical shifts (*δ*) are expressed in parts per million (ppm), with tetramethylsilane (TMS) as the internal standard, and chemical shifts are expressed in units of 10^−6^ (ppm) (See Supplementary Materials). All reagents used in the experiments are commercially available and analytically pure.

Preparation of (2*R*)-2-acetoxybutyric acid:

D-2-aminobutyric acid 1.0 kg (9.7 mol), sodium nitrite 2.68 kg (38.8 mol), and 10 L of acetic acid were added in batches at room temperature for approximately 1 h. After addition, the reaction was continued at room temperature for 3 h. Upon reaction by TLC, the mixture was filtered. And then, the filtrate was concentrated until no distillate remained, stirred with 2 L of dichloromethane for 1 h, and filtered. The filtrate was concentrated to obtain 1032.99 g of yellow oily product with a yield of 72.87%.

^1^H NMR (400 MHz, CDCl_3_-*d*_6_)δ: 10.51 (s, 1H), 4.99–4.96 (q, 1H), 2.16 (s, 3H), 2.01–1.84 (m, 2H), 1.05–1.01 (t, 3H).

ESI-MS:*m*/*z* = 147.2(C_6_H_9_O_4_ + H)^+^.

Preparation of (2*R*)-1-((4-carbamoyl-3-fluorophenyl)amino)-1-oxobutan-2-yl acetate:

(2*R*)-2-acetoxybutyric acid 496.8 g (3.4 mol) was dissolved in tetrahydrofuran 9.6 L, stirred, and replaced with N2 three times. After that, 308.0 g (2.0 mol) of 4-amino-2-fluorobenzene-1-carboxamide was added and cooled to below 0 °C. Then, pyridine 791 g (10.0 mol) was added. Finally, 1.27 kg (4.0 mol) of 1-propyl phosphoric anhydride (50% ethyl acetate solution) diluted with 200 mL of tetrahydrofuran was added dropwise to the reaction solution. After that, the solution was stirred at 0–5 °C for 10 min and then stirred at room temperature for a reaction for 1 h. Upon completion of the reaction, H_2_O was added to terminate the reaction and then extracted with EA. The organic phase was sequentially washed with 5% lemon acid, saturated sodium bicarbonate, H_2_O, and saturated salt water, and it was dried with anhydrous sodium sulfate and filtered in the end. Then, the solvent was evaporated to obtain the crude product. A total of 4 L of EA:MTBE (1:3) was added to the crude product, heated to 60 °C, and cooled down to 20–30 °C. Then, the solvent was filtered with suction to obtain a solid which was washed with n-heptane and dried by vacuum. Eventually, 485.0 g of white solid was obtained with a yield of 85.90%, purity of 98.80%, and an ee value of 99.07%.

ESI-MS:*m*/*z* = 283.1(C_13_H_15_FN_2_O_4_ + H)^+^.

Preparation of 2-fluoro-4-(((2*R*)-2-hydroxy-1-oxobutyl)amino)benzene-1-carboxamide:

(2*R*)-1-((4-carbamoyl-3-fluorophenyl)amino)-1-oxobutan-2-yl acetate 450.0 g (1.59 mol) was dissolved in a mixed solvent of 3 L of methanol and 4.5 L of H_2_O. Then, sodium carbonate 505.6 g (4.77 mol) was added and stirred at room temperature overnight. After the completion of the reaction by TLC was monitored, 6 L of H_2_O was slowly added to terminate the reaction. A large amount of white solid precipitated from the system. A total of 15 L of H_2_O was added continuously and then stirred for 2 h. The solution was filtered to obtain a solid which was dried by vacuum. Finally, 353.70 g of white solid was obtained with a yield of 92.60%, purity of 96.91%, and an ee value of 99.15%.

ESI-MS:*m*/*z* = 241.1(C_11_H_13_FN_2_O_3_ + H)^+^.

Preparation of (2*R*)-1-((4-carbamoyl-3-fluorophenyl)amino)-1-oxobutan-2-yl 4-methyl benzenesulfonate:

2-fluoro-4-(((2*R*)-2-hydroxy-1-oxobutyl)amino)benzene-1-carboxamide 344.0 g (1.43 mol) was dissolved in 4 L of dichloromethane and 1.2 L of acetonitrile. The triethylamine 289.2 g (2.86 mol) and DMAP 35 g (286 mmol) were added. Finally, at around 20 °C, a solution of P-Methylbenzenesulfonyl chloride 301.2 g (1.58 mol) in 2 L of dichloromethane and 400 mL of acetonitrile was added dropwise at around 20 °C, and the stirring was kept at 30 °C for a reaction overnight. After the completion of the reaction by TLC was monitored, 8 L of H_2_O was slowly added to terminate the reaction. The organic phase was extracted with 3 × 4 L dichloromethane. Then, the resulting solution was dried and concentrated to obtain the crude product. This crude product was dissolved in about 3.2 L of ethyl acetate and then cooled to crystallize to obtain 512.10 g of light yellow solid, with a yield of 90.8%, purity of 98.82%, and an ee value of 98.76%.

ESI-MS:*m*/*z* = 395.1(C_18_H_19_FN_2_O_5_S + H)^+^.

Preparation of target compound BAY2433334:

4-(5-chloro-2-[4-(trifluoromethyl)-1,2,3-triazol-1-yl)phenyl)-5-methoxy-1,2-dihydropyridin-2-one 500 g (1.35 mol) was added into a reactor, dissolved, and stirred in 1,4-dioxane 12 L. Then, opotassium carbonate 375 g (2.71 mol) and tetramethylguanidine (TMG) 155 g (1.35 mol) were added and stirred for 5 min. Then, (2*R*)-1-((4-carbamoyl-3-fluorophenyl)amino)-1-oxobutan-2-yl 4-methyl benzenesulfonate 512.0 g (1.30 mol) was added. The resulting solution was heated to 35 °C and stirred for a reaction until TLC monitoring was completed. The solution was cooled down to 10 ± 5 °C, 9 L of H_2_O and 5 L of isopropyl acetate were added, it stirred for 10–15 min, and then it was allowed to stratify. A total of 2 L of isopropyl acetate was added to the aqueous phase for back-extraction, stirred for 10–15 min, and allowed to stratify again. The organic phases were pooled, and then 6 L of H_2_O was added, washed, and stirred for 10–15 min, then allowed to stratify. The organic phase was concentrated at 55 ± 5 °C to 1.5 L, and 1 L of isopropyl acetate was added. The solution was concentrated continuously to 1.8 L, at which the concentration was terminated. The solution was stirred at 20 ± 5 °C for 10 h and then stirred at 10 ± 5 °C for 2 h. The solution was filtered with suction. Then, the wet product was removed and dried at 35 °C with blowing air for 18 h. After that, it was dried at 70 °C with blowing air for 24–48 h. The drying ended when moisture content < 2.0%. Then, 740.83 g of white solid was obtained with a yield of 96.11%, purity of 98.57%, and an ee value of 99.32%.

ESI-MS:*m*/*z* = 593.2(C_26_H_21_ClF_4_N_6_O_4_ + H)^+^.

^1^H NMR (400 MHz, DMSO-*d*_6_) *δ*: 10.79 (s, 1H), 9.14 (s, 1H), 7.87–7.79 (m, 3H), 7.71–7.63 (m, 2H), 7.57 (s, 1H), 7.54 (s, 1H), 7.39–7.36 (dd, 1H), 7.14 (s, 1H), 6.54 (s, 1H), 5.55–5.51 (m, 1H), 3.26 (s, 3H), 2.16–2.04 (m, 2H), 0.81–0.77 (t, 3H).

The specific data of the ^1^H NMR spectra of BAY2433334 are shown in Table 7.


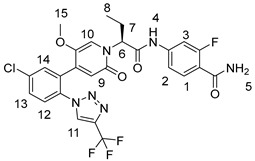


**Table 7:** Assignment of ^1^H NMR spectra of BAY2433334.

δppm	Multiplicity	Proton Number	Assignment
10.79	s	1	H-4
9.15–9.14	d	1	H-11
7.87–7.81	m	2	H-1, H-3
7.79	d	1	H-2
7.71–7.67	t	1	H-12
7.67–7.63	dd	1	H-14
7.57	s	1	H-5
7.54	s	1	H-5
7.39–7.36	dd	1	H-13
7.14	s	1	H-10
6.54	s	1	H-9
5.55–5.51	m	1	H-6
3.26	s	3	H-15
2.16–2.04	m	2	H-7
0.81–0.77	t	3	H-8

## 5. Conclusions

The synthetic process route of FXIa inhibitor BAY2433334 was studied and optimized in this paper. In the finally adopted Route 4, (2*R*)-2-aminobutyric acid was used as the raw material, and the process and post-treatment studies were conducted on the critical intermediate p. Intermediate p with high yield, high purity, and a high ee value was obtained, thereby avoiding the chiral resolution of the target product BAY2433334 and reducing the production cost. At the same time, this route can significantly reduce the proportion of O-alkylation byproducts, which improves product quality while further reducing production costs. Compared with the original route, the reagents are inexpensive and readily available, the reaction conditions are mild, the operation is simple, the quality of the target product is better, and it is more suitable for scale-up production.

## Figures and Tables

**Table 1:** Results of reaction condition optimization for intermediate O.

	D-2-Aminobutyric Acid	HOAc	NaNO_2_	H_2_O	Reaction Results
1	10 g	30 *V*	1.75 eq	/	5.2 g, yield: ~25%
2	10 g	20 *V*	2.0 eq	4 *V*	6 g, yield: ~43%
3	10 g	10 *V*	2.0 eq	4 *V*	5.8 g, yield: ~41%
5	10 g	10 *V*	2.0 eq	/	8.5 g, yield: ~60%
6	10 g	10 *V*	4.0 eq	4 *V*	7.6 g, yield: ~54%
7	10 g	10 *V*	4.0 eq	/	7.8 g, yield: ~55%
8	100 g	10 *V*	4.0 eq	/	106.3 g, yield: ~75% (removal of the water washing operation)

**Table 2:** Results of the preparation process study of intermediates.

Raw Material c	Intermediate q	Solvent	Condensing Agent and Catalyst	Base	Reaction Results
1 g	1.0 eq	10 *V* THF	CDI 1.2 eq	/	Poor purity of reaction system
2 g	1.7 eq	10 *V* DCM	EDCI 1.4 eq	N-Methylmorpholine 2.0 eq	Poor purity of reaction system
1.5 g	1.7 eq	10 *V* DCM DMF 0.01 eq THF 10 *V*	Oxalyl chloride 2.0 eq	TEA 2.0 eq	Crude: 2.4 g; yield: 80%
2 g	1.2 eq	10 *V* DCM DMF 0.01 eq THF 10 *V*	Oxalyl chloride 2.0 eq	TEA 2.0 eq	Large amount of raw material remains
2 g	1.5 eq	10 *V* DCM DMF 0.01 eq THF 10 *V*	Oxalyl chloride 2.0 eq	TEA 2.0 eq	Approximately 10% of the raw material remained
50 g	1.7 eq	10 *V* DCM THF 10 *V* DMF 0.01 eq	Oxalyl chloride 2.0 eq	TEA 2.0 eq	A small amount of raw material remained; the yield of crude product after purity correction was 80% but purification was not possible
5 g	1.7 eq	30 *V* THF	T3P 2.0 eq	Pyridine 5.0 eq	Yield: ~42%
1.0 g	1.7 eq	20 *V* THF	T_3_P 2.0 eq	Pyridine 5.0 eq	Chiral purity after EA hot slurry;yield after purity correction: ~66%
15.4 g	1.7 eq	20 *V* THF	T3P 2.0 eq	Pyridine 5.0 eq	After purification with mixed solvent of MTBE:EA, yield was 87% and ee value was 99.11%

THF: Tetrahydrofuran; CDI: 1,1′-Carbonyldiimidazole; DCM: Dichloromethane; EDCI: *N*-(3-dimethylaminopropyl)-*N*’-ethylcarbodiimide hydrochloride; DMF: *N*,*N*-Dimethylformamide; TEA: Triethylamine; T3P: Propyl phosphoric anhydride; EA: ethyl acetate; MTBE: tert-Butyl methyl ether.

**Table 3:** Investigation of base types in the preparation of intermediate q.

	Base	Reaction Results
1	Potassium carbonate	After the raw material was completely consumed, the crude intermediate q had a purity of 91.45%.
2	Cesium carbonate	After the raw material was completely consumed, the crude intermediate q had a purity of 85.09%.
3	Sodium carbonate	After the raw material was completely consumed, the crude intermediate q had a purity of 97.723%.

**Table 4:** Investigation of reaction temperature in the preparation of intermediate v.

	Temperature	Remaining Amount of Intermediate q
1	50 ± 5 °C	0.2%
2	30 ± 5 °C	0.1%
3	20 ± 5 °C	0.6%
4	10 ± 5 °C	6.6%
5	0 ± 5 °C	55.3%

**Table 5:** Investigation of initial drying temperatures for wet products.

	Initial Drying Temperature	Phenomena
1	70 °C	Melting and agglomeration of the wet product
2	50 °C	Partial melting and agglomeration of the wet product
3	30–40 °C	No melting or agglomeration

**Table 6:** Advantages of comparing Route 4 with route of CN111770917.

	N-alkylation/O-alkylation Ratio	ee Value of Crude Product (%)	Total Yield (%)
Route 4	35–45:1	95–97%	40–50%
CN111770917	9–10:1	85–93%	20–25%

## Data Availability

Data are contained within the article and Supplementary Materials.

## Supplementary Materials

The following supporting information can be downloaded at: https://www.mdpi.com/article/10.3390/molecules29246039/s1.
