# Supplementary material for: Study on Preparation Process of Anticoagulant BAY2433334
Source: Molecules. 2024 Dec 21;29(24):6039. doi: 10.3390/molecules29246039 (PMC11679754; doi:10.3390/molecules29246039)

Supporting Information

Study on Preparation Process of Anticoagulant BAY2433334

Yanqun Zeng[#, *], Guodong Cen[#], Guanglin Zhou, Xucheng Zu, Long Huang, Xiaoyu Wang

Chengdu shibeikang Biomedical Technlogy Co.,ltd, 26-1-2, No.2 Tianyu Road, Chendu Gaoxin west District, China

[#] Co-first author

[*] Correspondence: zengyq121@163.com

BAY2433334 ^{1}H-NMR

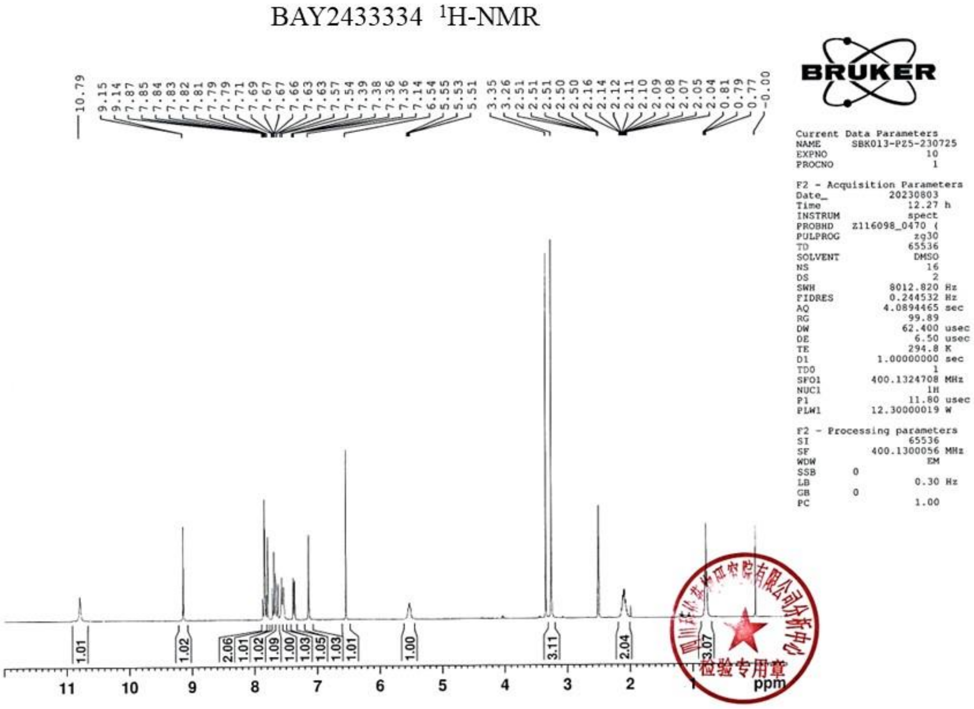

BAY2433334 LCMS

MS Report from Instrument: Agilent-LCMS-002

Ret. Time: 2.12 <<<< POSITIVE SPECTRA >>>>

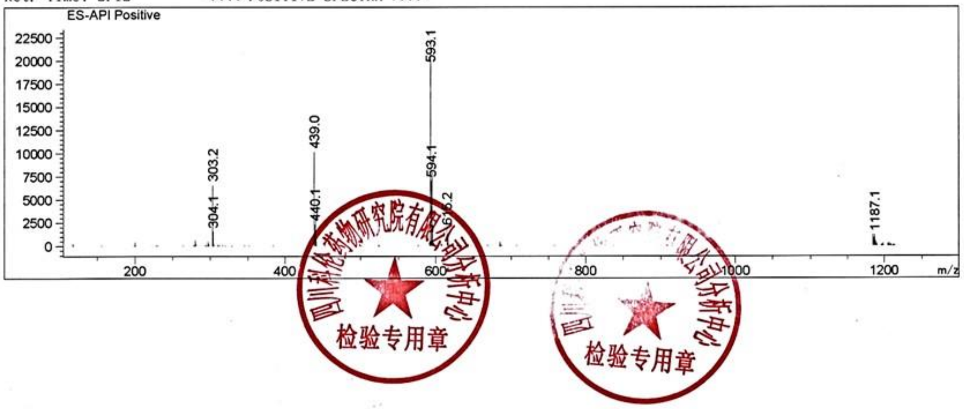

Compound O ^{1}H-NMR

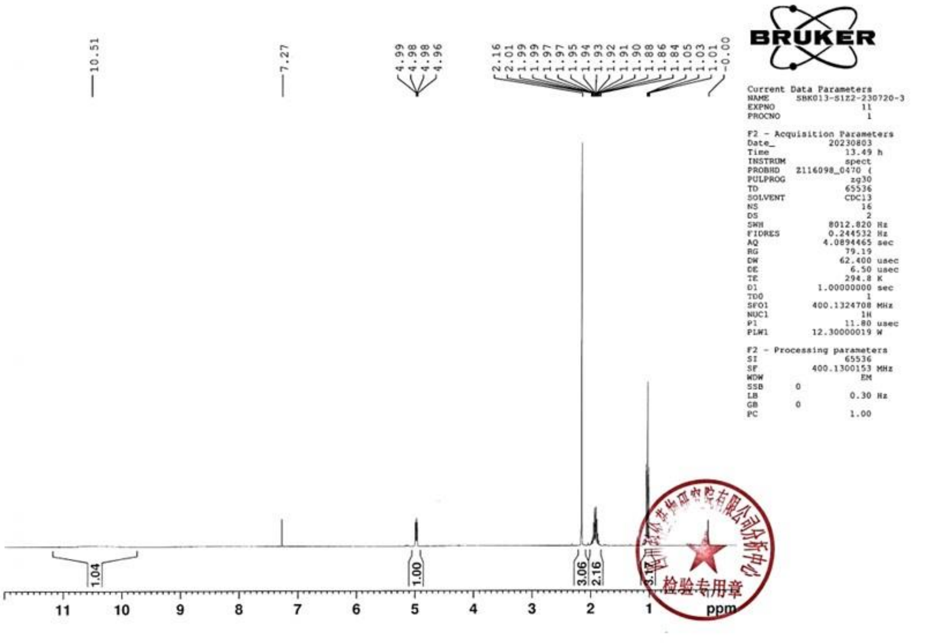

Compound P LCMS

MS Report from Instrument: Agilent-LCMS-002

Ret. Time: 0.92 <<<< POSITIVE SPECTRA >>>>

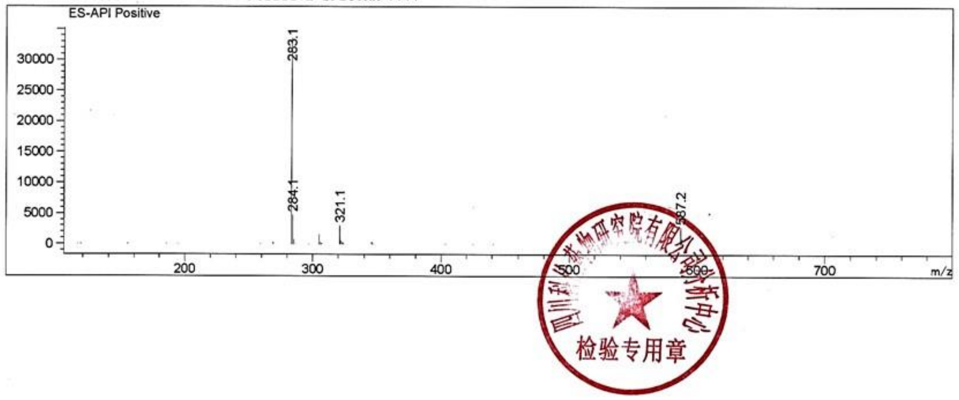

Compound q LCMS

MS Report from Instrument: Agilent-LCMS-002

Ret. Time: 0.12 <<<< POSITIVE SPECTRA >>>>

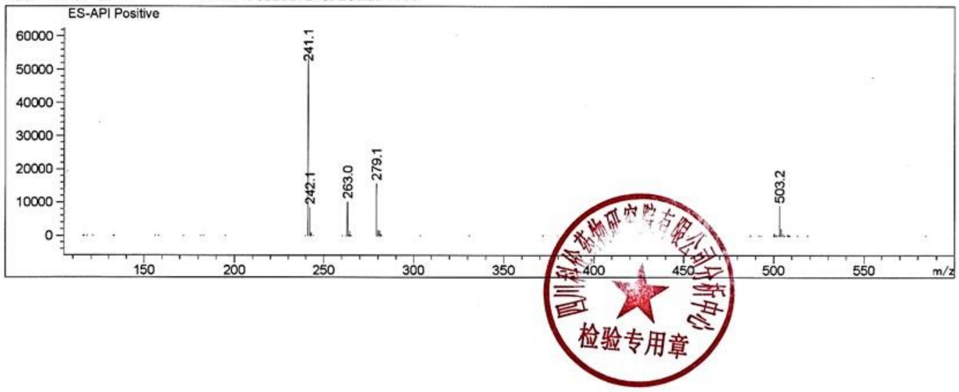

Compound v LCMS

MS Report from Instrument: Agilent-LCMS-002

Ret. Time: 1.22 <<<< POSITIVE SPECTRA >>>>

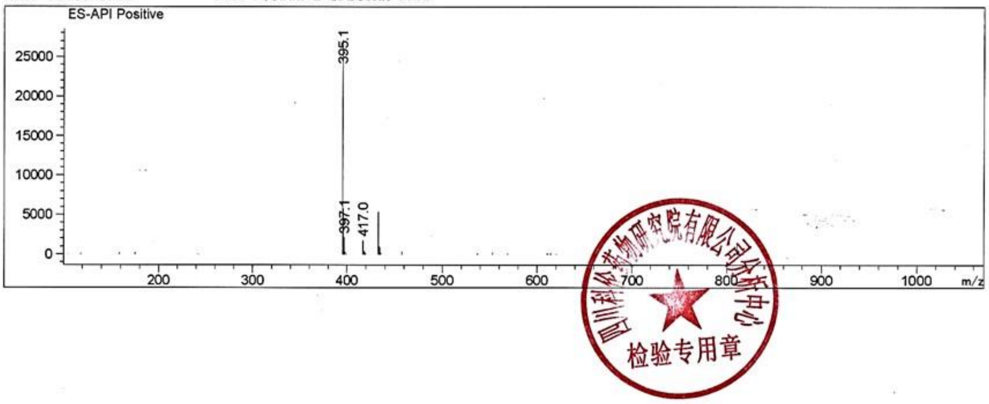

Supplement: Supplementary file 1 [file molecules-29-06039-s001.zip › molecules-3325854-supplementary/molecules-3325854-supplementary.pdf]
